# Predictors of Radiological and Clinical Disease Reactivation in Multiple Sclerosis Patients After Cessation of Natalizumab (Tysabri)

**DOI:** 10.1002/brb3.70904

**Published:** 2025-09-21

**Authors:** Roba El Zibaoui, Nabil K. El Ayoubi, Samia J. Khoury

**Affiliations:** ^1^ School of Medicine American University of Beirut Beirut Lebanon; ^2^ Department of Neurology American University of Beirut Medical Center Beirut Lebanon; ^3^ Nehme and Therese Tohme Multiple Sclerosis Center American University of Beirut Medical Center Beirut Lebanon

**Keywords:** disease reactivation | monoclonal antibodies | multiple sclerosis | natalizumab | Tysabri

## Abstract

**Background and Purpose**: Natalizumab (NTZ) is widely used for patients with highly active relapsing–remitting multiple sclerosis (RRMS), but its discontinuation can lead to disease reactivation, significantly impacting Middle Eastern populations. This study aimed to assess the frequency of disease reactivation post‐NTZ discontinuation and identify possible demographic predictors.

**Methods**: In this retrospective single‐center study, 171 patients with RRMS who received at least six consecutive NTZ infusions were analyzed. Clinical and radiological data were sourced from the AMIR registry. Binary and multivariate regression analyses were performed to identify factors associated with reactivation risk after 1 year, with a subset undergoing brain magnetic resonance imaging (MRI) segmentation and volumetric assessments.

**Results**: Among 92 eligible patients, 16 (17%) showed disease reactivation. Prolonged washout periods (>3 months) significantly increased reactivation risk (hazard ratio [HR] 1.015, 95% CI 1.007–1.022, *p* < 0.001), whereas higher Expanded Disability Status Scale (EDSS) scores correlated with a reduced risk (HR 0.492, 95% CI 0.280–0.866, *p* < 0.014). In the subset of cases that underwent brain MRI segmentation, higher NTZ infusion numbers and delayed bridging therapy initiation were linked to increased reactivation risk.

**Conclusion**: NTZ discontinuation requires thorough clinical evaluation for bridging therapy, yet optimal strategies remain undefined. Early bridging therapy initiation may mitigate reactivation, highlighting the need for further research on predictive factors and disease‐modifying therapy efficacy.

## Graphical abstract

This retrospective single‐center study looks at the frequency and degree of reactivation of disease following discontinuation of natalizumab in Lebanese patients with relapsing‐remitting multiple sclerosis, highlighting importance of early initiation of bridging disease‐modifying therapy. It also explores demographic predictors of reactivation to inform safer and more efficient transition practices in resource‐limited settings.

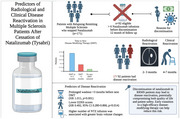

**Abbreviations**
BMI: body mass index
CNS: central nervous system
DMT: disease‐modifying therapies
EDSS: Expanded Disability Status Scale
Gd+: gadolinium‐enhancing
GM: grey matter
JC virus: John Cunningham virus
MRI: magnetic resonance imaging
MS: multiple sclerosis
NEDA: no evidence of disease activity
NTZ: natalizumab
PML: progressive multifocal leukoencephalopathy
RRMS: relapsing‐remitting multiple sclerosis

## 1 Introduction

Natalizumab (NTZ) is a highly specific α4‐integrin antagonist, disease‐modifying agent that has been shown in several large randomized controlled studies (AFFIRM, SENTINEL) and real‐world observations to effectively reduce disease activity in relapsing–remitting multiple sclerosis (RRMS) by lowering relapse rates and improving radiological findings on magnetic resonance imaging (MRI) (Delbue et al. 2017; Clerico et al. 2017). Notably, early treatment initiation, within 1 year of symptom onset, has been associated with considerable reductions in Expanded Disability Status Scale (EDSS) scores and confirmed disability improvement, as observed in the TOP study (Wiendl et al. 2021). These findings suggest that early NTZ initiation during the inflammatory phase of multiple sclerosis (MS), when axonal dysfunction is more reversible, may maximize neurological recovery and long‐term functional outcomes (Wiendl et al. 2021).

In addition to reducing clinical and radiological disease activity, NTZ also exerts regionally specific brain volume effects. Inflammation reductions early in the course, especially in white matter regions like the corpus callosum and cerebellum, can cause transient “pseudoatrophy,” reflecting decreased edema, rather than true tissue loss (Rekik et al. 2022). Long‐term, NTZ has been found to slow atrophy in both subcortical and cortical grey matter (GM), with some studies even reporting volume expansion in deep GM structures like the thalamus (Rekik et al. 2022). This suggests a function for NTZ in preserving cognitive ability and structural integrity in areas involved in processing and memory (Rekik et al. 2022). However, the effects of NTZ discontinuation on brain volume and cognitive function during disease reactivation remain uncertain (Wiendl et al. 2021).

Despite these benefits, withdrawal of NTZ is often necessary in clinical practice, most commonly in patients previously exposed to John Cunningham virus (JC virus) who are at high risk of progressive multifocal leukoencephalopathy (PML), pregnancy, or logistical and financial considerations (Wingerchuk and Carter 2014; Cofield et al. 2016). However, NTZ withdrawal carries a well‐documented risk of disease reactivation due to lymphocyte migration through the blood–brain barrier, leading to heightened inflammatory activity in the central nervous system (CNS) (Auer et al. 2021; Plavina et al. 2017). This phenomenon typically peaks 8–12 weeks after stopping the drug, mirroring the drug's pharmacokinetic and pharmacodynamic properties. This reactivation can lead to worsening EDSS scores, reduced quality of life, increased healthcare utilization, and reduced work productivity (Prosperini et al. 2019).

A “washout” period between NTZ and other DMTs has been employed traditionally to reduce overlapping immunosuppressive activity and PML risk (Naismith 2022). However, this strategy has been associated with increased risk of rebound disease activity, particularly if followed by a switch to lower‐efficacy drugs such as interferon beta, glatiramer acetate, or teriflunomide as opposed to a high‐ or moderate‐efficacy treatment (Hersh et al. 2020). In light of the growing awareness of risk of disease reactivation and increased focus on achieving no evidence of disease activity (NEDA‐3), there is a growing necessity for effective strategies to minimize reactivation and prevent worsening outcomes (Pappolla et al. 2024; Capobianco et al. 2015; O'Connor et al. 2011; Sorensen et al. 2014; Lo Re et al. 2015). Furthermore, predicting future disease activity is critical for guiding therapy decisions, yet uncertainties remain about whether factors like demographics, prior relapses, and treatment history influence the likelihood and intensity of disease reactivation (Prosperini et al. 2019).

In the Middle East, the incidence and prevalence of MS have risen notably during the last decades, predominantly in females. In Lebanon specifically, the lack of national registries has rendered the precise prevalence and incidence undetermined; however, existing epidemiological research has classified it as a moderate‐to‐high‐risk area for MS, consistent with broader regional trends (Zeineddine et al. 2021). The rising burden of MS in the region reinforces the need to address these gaps to identify individuals at risk of disease activation, optimize treatment transitions, minimize productivity losses, and improve long‐term outcomes in patients with active RRMS, particularly amidst Lebanon's ongoing economic crisis that has intensified healthcare resource shortages (Dahham et al. 2023). Therefore, the primary objective of this study is to investigate the rate and severity of disease reactivation following NTZ cessation in a Lebanese cohort of RRMS patients, along with assessing the impact of time to initiation of the next DMT. A secondary objective is to ascertain whether demographic factors are predictive of risk of reactivation, for the ultimate goal of guiding safer, more efficacious NTZ transition approaches in real‐world, resource‐constrained clinical practice.

## 2 Methods

### 2.1 Definition of Cases

**FIGURE 1:**
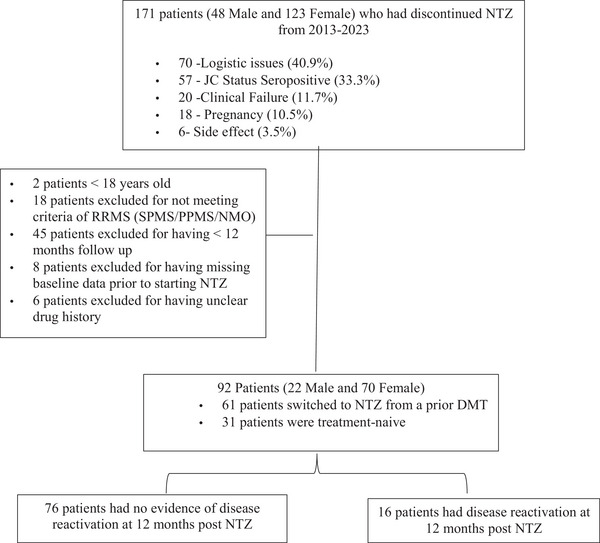
The selection of the study cohort. DMT, disease‐modifying therapy; NMO, neuromyelitis optica; NTZ, natalizumab; PPMS, primary progressive MS; RRMS, relapsing‐remitting multiple sclerosis; SPMS, secondary progressive MS.

We conducted a retrospective, single‐center study at the Nehme and Therese Tohme Multiple Sclerosis Center at the American University of Beirut Medical Center based on the AUBMC Multiple Sclerosis Interdisciplinary Research (AMIR), the largest MS database in Lebanon. AMIR is a single‐center longitudinal prospective study that has been ongoing since 2012 and has enrolled approximately 3000 MS patients diagnosed per the McDonald criteria. Patients are directly recruited from clinical practice and monitored over time with standardized clinical evaluations every 6 months, as well as annual or bi‐annual MRIs and quality of life surveys. The majority of patients choose to take part in the biobank for blood, DNA, and urine annual collection (Bove et al. 2017). Patients can also participate in annual longitudinal optical coherence tomography (OCT) assessments. This registry includes patients with all types of MS, such as CIS, RIS as well as those with NMO. Clinical, demographic, and radiological data from medical records of patients diagnosed with RRMS who had ever received and stopped treatment with NTZ were collected using electronic medical records through EPIC and Redcap. Data were collected on all patients over the age of 18 without a history of recent moderate‐to‐severe traumatic brain injury, uncontrolled cerebrovascular disorder, or neurological malignancy, currently diagnosed with RRMS as per 2017 McDonald's criteria (Polman et al. 2011). Patients met the inclusion criteria if they had received at least six consecutive infusions of NTZ before discontinuation. Discontinuation was defined as 3 months without any NTZ infusions (Lo Re et al. 2015). Patients were selected only if they had clinical and radiological data available for at least 12 months after their last infusion. We identified 171 MS patients who had discontinued NTZ treatment between 2013 and 2023, of whom 92 met our inclusion criteria. A flowchart displaying our patient selection of the study cohort is shown in Figure 1.

### 2.2 Data Collection

Patient demographic characteristics, including sex, smoking status, comorbidities, body mass index (BMI), age of MS diagnosis, and family history of MS, were recorded. Moreover, information on any history of prior DMT use, age at NTZ initiation, the number of infusions received, and average vitamin D levels while on NTZ was also collected. Clinically meaningful performance benchmarks, including the 25‐foot walk test, 9‐hole peg test, and Symbol Digits Modalities Test, were collected in addition to the calculated EDSS score before initiation of NTZ, during NTZ therapy, and up to 1 year following NTZ discontinuation (Koch et al. 2021). All relapses before therapy, along with any evidence of disease reactivation while on treatment, were noted. Analysis of MRIs consisted of T1‐weighted and T2‐weighted images with and without gadolinium contrast agents. The percentage of brain volume change was also recorded before and after NTZ therapy. The number of brain lesions detected was subsequently categorized (mild: 1–5 lesions; moderate: 5–15 lesions; severe: >15 lesions). Any new changes in Gd‐enhancing T1 lesions, T2‐hyperintense lesion volume, and new and enlarging T2‐hyperintense lesions were recorded and confirmed by a neurologist (Prosperini et al. 2019). Data were collected about the use of steroids during relapse, in addition to the type and timing of new DMT. Disease reactivation was defined clinically as new symptoms, or exacerbation of existing symptoms in the absence of a concurrent illness, or radiologically with new Gd‐enhanced lesions or T2 lesions on MRI, or evidence of worsening EDSS scores at 6 and 12 months post‐NTZ as defined by Sharmin et al. (2022).

MRI was used to provide quantitative information about lesion number and volume. The MRI protocol utilized a 3 T Philips Ingenia system, acquiring two 3DT1 turbo field echo sequences with gadolinium (Darwish et al. 2020). Lesion volume was determined through manual tracing and semiautomatic detection (Darwish et al. 2020). Multiparametric segmentation integrates multiple sequences for accurate measurements, with tools like SIENAX estimating tissue volumes and VolBrain segmenting thalamus regions (Darwish et al. 2020). The computed brain and internal structure volumes were then normalized by the brain volume. For this study, we looked at GM, white matter, cerebrospinal fluid (CSF), and deep GM volume.

### 2.3 Ethical Approval

This study was approved by the Institutional Review Board of the American University of Beirut under the AMIR registry for data use in the “Comprehensive Multiple Sclerosis Database” protocol IM.SK1.01. All participants provided written informed consent prior to data collection, and the study was conducted in accordance with the relevant guidelines and regulations.

### 2.4 Statistical Analysis

Quantitative variables were expressed as mean (standard deviation); qualitative variables were expressed as proportions. A univariate Cox regression analysis was first used to identify individual variables associated with the risk of reactivation at 12 months of follow‐up post‐NTZ. A multivariable regression model included variables with a statistically significant association on univariate analysis. All analyses were carried out using SPSS software Version 24.0.

## 3 Results

### 3.1 Patient Demographics

A total of 92 patients were included in the study. The patients received a mean number of 29.2 (range 3–86, SD ± 18.3) infusions of NTZ. The clinical characteristics of the study population are displayed in Table 1. NTZ was used as a first‐line therapy in 33.7% (*n* = 31) of patients and was initiated within 12 months of the first symptoms. Pre‐NTZ, patients in our cohort had a mean of 3.3 relapses (range 0–12, SD ± 2.3). In the pre‐NTZ MRI scan, a substantial proportion of the study cohort, 41.3% of the patients (*n* = 38), had a severe lesion load defined as having more than 15 lesions. Only six patients (6.5%) had an EDSS of 5.5 or higher at the start of NTZ treatment. The treatment was well tolerated by most patients, with only six patients (6.5%) discontinuing treatment due to adverse events. Additionally, only 10 patients (10.9%) experienced a relapse while on treatment. Although on treatment, patients had very low radiological activity with an average of <1 Gd‐enhancing lesions and <2 T2 new non‐enhancing lesions on MRI. The most common reason for NTZ withdrawal was due to logistic issues in obtaining or financing the medication (42.4%) as opposed to the JC virus seropositivity/risk of PML (31.5%). All reasons for treatment discontinuation are displayed in Table 1.

**TABLE 1:** Demographic and clinical characteristics of the study population.

Variable	All patients (*n* = 92)	Disease reactivation (*n* = 16)	No disease reactivation (*n* = 76)	*p* values
Female gender, *n* (%)	70 (76.1)	14 (87.5)	56 (73.7)	0.39
Family history of MS, *n* (%)	19 (20.7)	2 (12.5)	17 (22.4)	0.58
Age at diagnosis of RRMS (years, mean ± SD)	27.7 ± 9.8	25.4 ± 8.7	28.2 ± 10.0	0.27
Age at NTZ initiation (years, mean ± SD)	32.3 ± 10.6	28.5 ± 8.9	33.1 ± 10.8	0.08
BMI (mean ± SD)	24.1 ± 4.1	23.1 ± 4.3	24.4 ± 4.1	0.28
Vitamin D (ng/mL) on NTZ (mean ± SD)	51.2 ± 18.9	44.8 ± 21.0	52.2 ± 18.6	0.31
No. of relapses before NTZ (mean ± SD)	3.3 ± 2.3	2.6 ± 1.4	3.6 ± 2.0	0.02*
No. of NTZ infusions (mean ± SD)	29.2 ± 18.3	34.1 ± 22.5	28.1 ± 17.3	0.33
**Smoking status, *n* (%)**				
Smoker	34 (37.0)	6 (37.5)	28 (36.8)	0.81
Non‐smoker	39 (42.4)	6 (37.5)	33 (43.4)	0.88
Ex‐smoker	12 (13.0)	3 (18.8)	9 (11.8)	0.74
Unspecified	7 (7.6)	1 (6.3)	6 (7.9)	0.77
**Brain MRI lesion load before NTZ, *n* (%)**				
Mild (1–5 lesions)	29 (31.5)	2 (12.5)	27 (35.5)	0.13
Moderate (6–15 lesions)	25 (27.2)	6 (37.5)	19 (25.0)	0.48
Severe (>15 lesions)	38 (41.3)	8 (50.0)	30 (39.5)	0.62
**EDSS scores (mean ± SD)**				
EDSS at NTZ initiation	2.4 ± 1.6	1.8 ± 1.4	2.6 ± 1.6	0.05*
EDSS at NTZ discontinuation	1.73 ± 1.6	0.86 ± 1.5	1.92 ± 1.5	0.02*
EDSS >12 months after NTZ	2.0 ± 1.6	2.69 ± 1.3	1.92 ± 1.5	0.08
**Primary reason for NTZ discontinuation, *n* (%)**				
Logistic issues	39 (42.4)	6 (37.5)	33 (43.4)	0.88
JC virus seropositive	29 (31.5)	6 (37.5)	23 (30.3)	0.79
Clinical failure	10 (10.9)	1 (6.3)	9 (11.8)	0.83
Pregnancy	8 (8.7)	2 (12.5)	6 (7.9)	0.91
Side effects of medication	6 (6.5)	1 (6.3)	5 (6.6)	0.61
**DMT initiation timing, *n* (%)**				
DMT initiated within 0–3 months	66 (71.7)	0 (0)	66 (86.8)	<0.001**
DMT initiated after >3 months	25 (27.2)	15 (93.8)	10 (13.2)	<0.001**
No new DMT initiated	1 (1.1)	1 (6.3)	0 (0)	<0.001**

*Note*: *p < 0.05, **p < 0.01 (*p* values <0.05 are considered statistically significant and are marked). Continuous variables are expressed as mean ± standard deviation (SD), whereas categorical variables are reported as counts (*n*) and percentages (%).

Abbreviations: BMI, body mass index; DMT, disease‐modifying therapy; EDSS, Expanded Disability Status Scale; JC virus, John Cunningham virus; MRI, magnetic resonance imaging; MS, multiple sclerosis; NTZ, natalizumab; RRMS, relapsing‐remitting multiple sclerosis.

### 3.2 Disease Reactivation after NTZ Discontinuation

**FIGURE 2:**
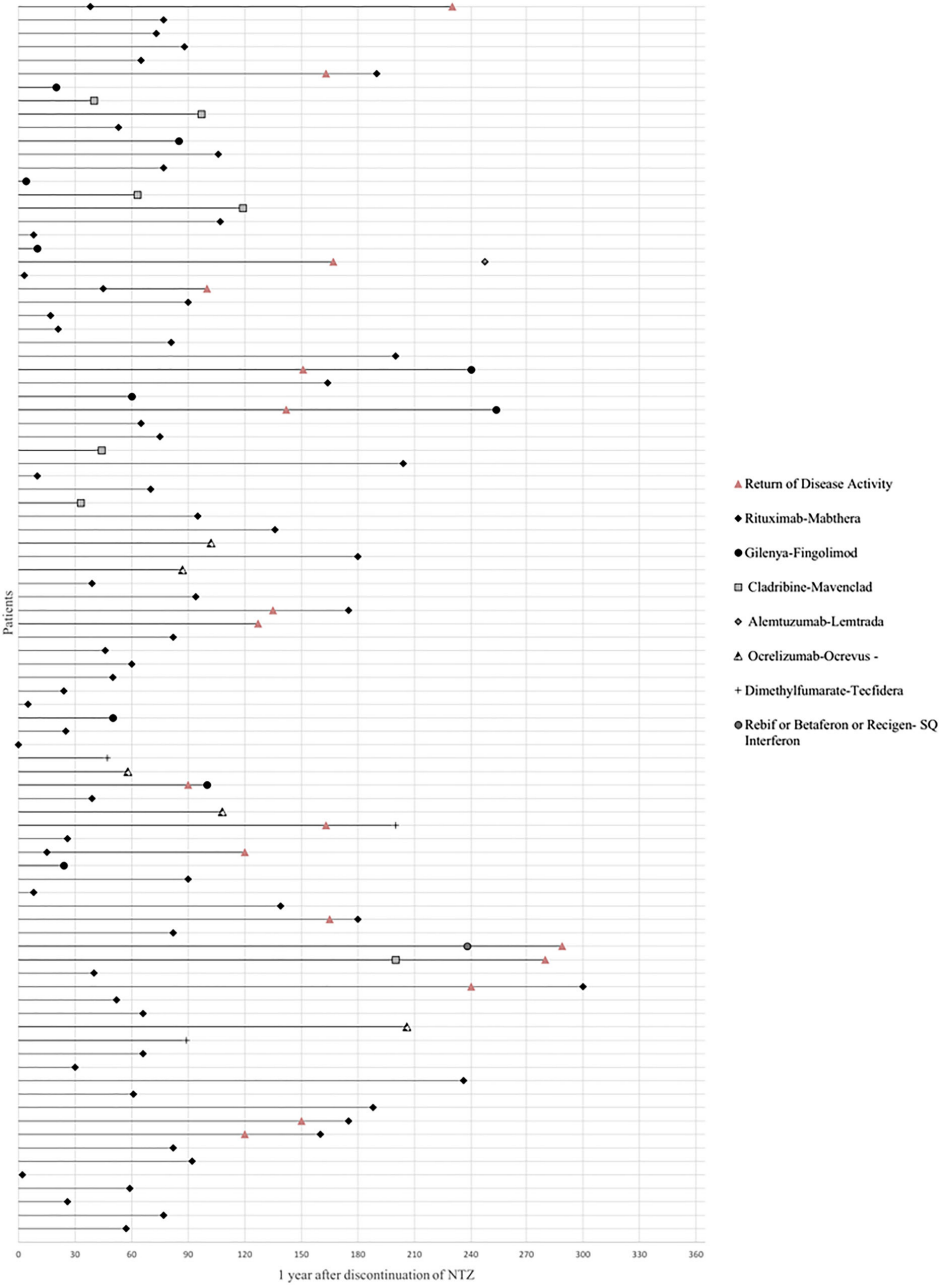
Horizontal lines represent time since NTZ cessation; symbols indicate therapy initiation and reactivation timing (clinical or radiological). One‐year post‐NTZ serves as a reference point. Treatments included rituximab (69.6%), fingolimod (10.9%), cladribine (7.6%), ocrelizumab (5.4%), dimethyl fumarate (3.3%), subcutaneous Interferon (1.1%), alemtuzumab (1.1%), and no new treatment (1.1%). DMT, disease‐modifying therapy; NTZ, natalizumab.

After discontinuation of NTZ, 16 patients (2 males [12.5%] and 14 females [87.5%]) had evidence of disease reactivation. Clinical reactivation peaked at approximately 4–7 months after the last infusion, whereas radiological reactivation was apparent even at 6–12 weeks of NTZ discontinuation. Subsequent DMT was initiated for nearly all patients, within 3 months for 71.7% (*n* = 66) and after 3 months for 27.2% (*n* = 25), respectively (Table 1). Only one patient did not receive any bridging DMT. The most commonly prescribed DMT was rituximab (69.6%). The time from NTZ discontinuation to initiation of the next disease‐modifying therapy (DMT) and timing of disease reactivation (clinical or radiological) is shown in Figure 2. A higher percentage of patients experienced disease reactivation when the washout period was longer than 3 months (Table 1). A total of 11 (68.75%) of 16 patients meeting the definition of disease reactivation received a course of corticosteroid treatment. The patients who had disease activation were younger, had a lower mean number of prior relapses, and had lower baseline EDSS scores when compared to patients who did not have reactivation (Table 1). Moreover, among the patients who had a relapse while on Tysabri, only one patient experienced disease reactivation after discontinuing the medication. The results of the regression analyses are shown in Table 2. A longer number of days before starting a new DMT was associated with a significantly higher risk for reactivation at 12 months (1.015, 95% CI 1.007–1.022, *p* < 0.001). Additionally, a higher EDSS score at the time of NTZ discontinuation was associated with a lower likelihood of disease reactivation (0.492, 95% CI 0.280–0.866 (*p* < 0.014).

**TABLE 2:** Statistical analysis of disease reactivation predictors after natalizumab (NTZ) using univariate and multivariate analysis.

Variable	Hazard ratio (HR)	95% CI (lower–upper)	*p* value
**Univariate analysis**			
Total days before new DMT	1.015	1.007–1.022	<0.001**
EDSS at NTZ discontinuation	0.492	0.280–0.866	0.014*
No. of relapses before NTZ	0.77	0.57–1.04	0.08
Age at initiation of NTZ	0.95	0.89–1.01	0.12
Baseline EDSS on NTZ	0.74	0.50–1.09	0.11
Age at diagnosis of RRMS	0.97	0.91–1.03	0.34
Duration of illness (years)	0.91	0.80–1.04	0.18
Brain MRI lesion load before NTZ	4.08	0.86–19.30	0.08
**Treatment‐related factors**			
Prior use of DMT	0.66	0.33–1.312	0.24
No. of NTZ infusions	1.02	0.99–1.045	0.24
**Demographic and lifestyle factors**			
Sex (male vs. female)	0.40	0.08–1.92	0.25
BMI	0.92	0.79–1.06	0.23
Smoking status (smoker vs. non)	1.20	0.34–4.07	0.80
Family history of MS	0.50	0.10–2.40	0.38
Vitamin D levels	0.98	0.95–1.01	0.27
**Multivariate analysis**			
Total days before new DMT	1.01	1.01–1.02	<0.001**
EDSS at NTZ discontinuation	0.49	0.20–0.90	0.025*

*Note*: *p < 0.05, **p < 0.01 (*p* values <0.05 are considered statistically significant and are marked).

Abbreviations: BMI, body mass index; CI, confidence intervals; DMT, disease‐modifying therapy; EDSS, Expanded Disability Status Scale; MRI, magnetic resonance imaging; MS, multiple sclerosis; NTZ, natalizumab; RRMS, relapsing‐remitting multiple sclerosis.

In the univariate analysis, age, gender, disease duration, BMI, smoking status, vitamin D level, prior use of DMT, number of prior relapses, and number of NTZ infusions were not associated with the risk of disease reactivation after NTZ was discontinued. Consequently, only significant variables were used in the multivariate analysis. In the multivariate analysis, both variables, “total days before new DMT” and “EDSS at NTZ discontinuation”, remained significant.

### 3.3 Percent Change in Brain Volume

**FIGURE 3:**
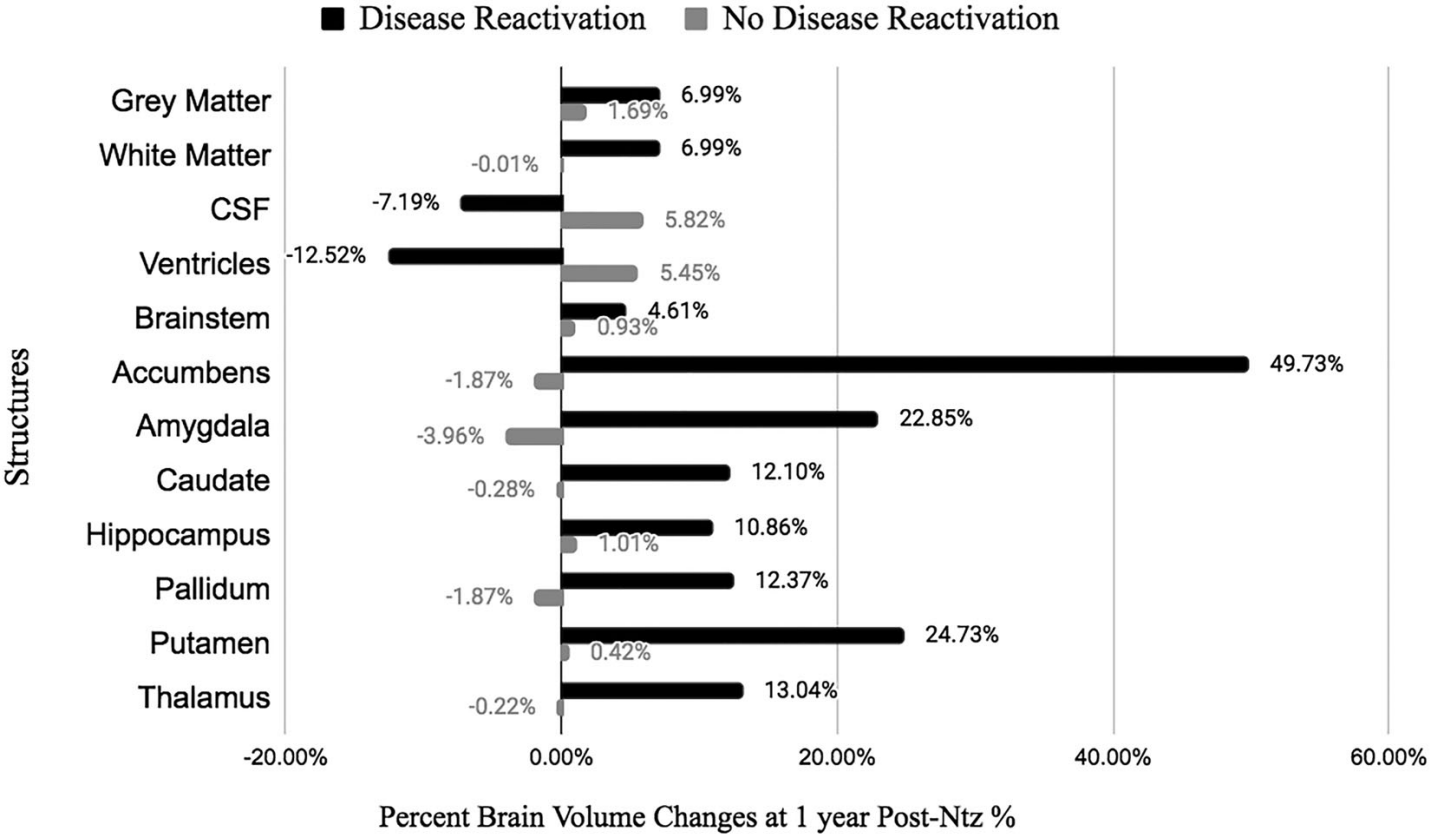
Changes in brain volume at 1‐year post NTZ. All brain volume measures were normalized. CSF, cerebrospinal fluid; NTZ, natalizumab.

MRIs according to MS protocol within pre‐NTZ and within 1‐year post‐NTZ discontinuation were available in a subset of 27 patients. We measured the percentage of brain volume change from baseline. The baseline characteristics are displayed in Table 3. The changes in brain volume are displayed in Figure 3. In patients who had experienced disease reactivation post‐NTZ, there was an increase in volume in white matter, GM, and subcortical structures, except for ventricles and CSF volume, when compared to patients who did not have disease reactivation.

**TABLE 3:** Clinical characteristics of the cohort subset with brain volume changes (*n* = 27).

Variable	Disease activation (*n* = 5)	No disease activation (*n* = 22)
Age at initiation of NTZ (mean ± SD)	26.4 ± 6.0	33.2 ± 7.4
Age at diagnosis of RRMS (mean ± SD)	24.2 ± 6.5	27.8 ± 7.8
No. of NTZ infusions (mean ± SD)	44.8 ± 26.6	25.4 ± 10.0
No. of relapses before NTZ (mean ± SD)	2.6 ± 1.5	3.2 ± 1.8
Baseline EDSS on NTZ (mean ± SD)	2.8 ± 0.4	2.45 ± 1.6
EDSS at NTZ discontinuation (mean ± SD)	1.0 ± 0.7	2.02 ± 1.5
Brain MRI lesion load before NTZ, *n* (%)		
Mild (1–5 lesions)	1 (20.0)	9 (40.9)
Moderate (6–15 lesions)	0 (0.0)	6 (27.3)
Severe (>15 lesions)	4 (80.0)	7 (31.8)

Abbreviations: EDSS, Expanded Disability Status Scale; MRI, magnetic resonance imaging; NTZ, natalizumab; RRMS, relapsing‐remitting multiple sclerosis; SD, standard deviation.

Subgroup analysis was done to identify individual variables associated with disease reactivation at 12 months post‐NTZ and is displayed in Table 4. Consistent with previous results, a longer number of days before starting a new DMT was associated with a significantly higher risk for reactivation at 12 months (1.015, 95% CI 1.010–1.046, *p* < 0.001). Additionally, a higher number of NTZ infusions was associated with a higher likelihood of disease reactivation (1.073, 95% CI 1.050–1.152, *p* < 0.050). No other predictive variables were associated with a statistically significant risk of disease reactivation.

**TABLE 4:** Statistical analysis with 95% confidence intervals for prognostic factors linked to disease reactivation post‐NTZ in the cohort subset with brain volume changes.

Variable	Hazard ratio	95% CI	*p* value
Total days before new DMT	1.02	1.01–1.05	<0.001**
No. of NTZ infusions	1.07	1.05–1.15	0.050*
Age at initiation of NTZ	0.86	0.73–1.02	0.08
Age at diagnosis of RRMS	0.93	0.81–1.08	0.34
No. of relapses before NTZ	0.82	0.46–1.46	0.09
Baseline EDSS on NTZ	1.19	0.61–2.33	0.62
EDSS at NTZ discontinuation	0.45	0.15–1.32	0.14
Prior use of DMT	0.46	0.10–2.04	0.31
Brain MRI lesion load before NTZ	2.77	0.26–29.05	0.40

*Note*: *p < 0.05, **p < 0.01 (*p* values <0.05 are considered statistically significant and are marked).

Abbreviations: CI, confidence intervals; DMT, disease‐modifying therapy; EDSS, Expanded Disability Status Scale; MRI, magnetic resonance imaging; NTZ, natalizumab; RRMS, relapsing‐remitting multiple sclerosis.

## 4 Discussion

In our study, 17% of RRMS patients had disease reactivation within 1 year after NTZ discontinuation. These results are consistent with reports from previous studies that have results that range from 13.5% to 58%, with variability likely due to differences in sample size and characteristics of study populations (Mustonen et al. 2020). Evidence of disease reactivation on MRI was evident at approximately 6–12 weeks after NTZ discontinuation, whereas clinical reactivation peaked at approximately 4–7 months after the last infusion, both of which are consistent with NTZ's pharmacological properties (Serra López‐Matencio et al. 2021). We demonstrated that a delay of more than 3 months in initiating another DMT was a statistically significant factor affecting disease reactivation. This finding can be explained by the rapid migration of circulating T cells, B cells, and monocytes into the CNS following NTZ discontinuation. Moreover, subtherapeutic alpha 4‐integrin saturation and upregulation of interleukins such as IL‐17 and IL‐22 often promote increased production of CD4 and CD8 T cells that may result in disease reactivation once NTZ is no longer present (Larochelle et al. 2017).

Additionally, a higher EDSS score at NTZ discontinuation was associated with a statistically significant decreased chance of disease reactivation. This is in contrast with earlier studies that reported that higher EDSS scores and EDSS worsening while on NTZ were both significant risk factors for both short‐term and long‐term disease reactivation and progression post‐NTZ (Prosperini et al. 2019; Vidal‐Jordana et al. 2015). On the other hand, other recent studies reported that patients with lower EDSS were at higher risk of disease reactivation (Zhang et al. 2022; Salhofer‐Polanyi et al. 2014). The explanation for these contrasting findings remains unclear. Nevertheless, these observations may suggest that the pathophysiologic correlates of the return of disease activity cannot be predicted solely based on the EDSS score.

We did not detect any significant association between demographic variables and disease reactivation. Previous studies had reported an association of younger age, higher and lower pre‐NTZ relapse rates, higher baseline EDSS scores, and fewer infusions of NTZ with higher reactivation rates (Prosperini et al. 2019; Lo Re et al. 2015; Mustonen et al. 2020; Hoepner et al. 2014). Although patients with evidence of disease reactivation were younger in our cohort, this did not reach statistical significance as reported in earlier studies (Lo Re et al. 2015; Salhofer‐Polanyi et al. 2014).

The loss of brain volume is one of the pathologic hallmarks of MS and is often correlated with measures of clinical disability and treatment responses while also being a predictor of future disability or disease progression due to its correlation with neuroaxonal damage (Nakamura et al. 2024; Richert et al. 2006). One of the well‐known transient changes of initiating NTZ is accelerated brain volume decrease. This “pseudoatrophy” effect is thought to reflect the hydrodynamic changes related to NTZ efficacy in decreasing inflammation, edema, and cellular infiltration rather than the loss of actual brain tissue (Richert et al. 2006). Consequently, it has been proposed that the discontinuation of NTZ could lead to an increase in brain volume due to a post‐NTZ surge of inflammatory cytokines (Nakamura et al. 2024). Our results support this hypothesis as we observed a significant increase in brain volume of patients experiencing disease reactivation. This likely correlates with the increased focal inflammatory activity. A univariate analysis found that patients with an increase in brain volume had received a greater number of infusions and were not bridged to another DMT within a 3‐month window period. Patients who received bridging therapy maintained a relatively constant or mild 1%–2% decrease in brain volume, consistent with reported levels of brain atrophy in MS (Mellergård et al. 2017).

Collectively, the results of this study and subgroup analysis support the use of a highly effective alternative DMT as bridging therapy within 3 months of NTZ discontinuation. This recommendation has been widely echoed and advocated across various studies to prevent or decrease the severity of disease reactivation (Ontaneda et al. 2019). The observed efficiency in the initiation of an alternative high‐efficacy DMT within a 2–4‐month window from NTZ discontinuation is by targeting the surge of peripheral immune cells and inflammatory cytokines expected to reach similar levels in untreated patients around 16 weeks post‐NTZ discontinuation (Prosperini et al. 2019). Nonetheless, there have been no established protocols for the timing and choice of the next DMT, which is reflected in the wide range of therapies, timing, and duration of interruption across various studies (Prosperini et al. 2019). The RESTORE trial showed that the risk of relapses and MRI activity remained among patients treated with interferon beta‐1a, glatiramer acetate, and steroids immediately after NTZ cessation (Fox et al. 2014). Similar results were also reported in other multicenter, observational, and retrospective analyses in which treatment interruption led to the recurrence of clinical and MRI activity despite alternative DMT or steroid administration (Prosperini et al. 2019).

Due to the limited efficacy of previous bridging therapy, the use of monoclonal antibody medications such as alemtuzumab, ocrelizumab, and rituximab has gained popularity and yielded optimistic outcomes (Fox et al. 2014; de Sèze et al. 2023; Zhu et al. 2023). In our study, the majority of patients were bridged to rituximab, anti‐CD20 monoclonal antibodies, whose efficacy is mediated by its ability to selectively deplete CD20+ B and CD20+ T cells and efficiently suppress inflammatory disease activity and reduce proliferation and proinflammatory cytokines Th1, Th17, and TNFα (Fox et al. 2014; de Sèze et al. 2023). Apart from its efficacy, the medication is also available, affordable, and has a favorable side effect profile. These findings are also supported by a recent Swedish study conducted at 3 MS centers that found the patients bridged to rituximab post‐NTZ were less likely to experience clinical relapse and fewer contrast‐enhancing lesions, even after adjusting for possible confounders (Alping et al. 2016). As the efficacy of other DMTs in preventing post‐NTZ disease reactivation is still being evaluated, close attention should be paid to making accurate estimates of safe untreated intervals for different therapies to minimize the risk of disease reactivation based on the known pharmacodynamics and pharmacokinetics of these drugs (Smoot et al. 2023; Roos et al. 2022).

The limitations of our study are mainly due to the low sample size and the lack of comprehensive MRI data for patients who discontinued NTZ. Moreover, due to the retrospective design of the study, we could not randomize or control for certain variables, such as types of alternative therapies used or washout schedules, which limits our ability to make any causal inferences about the impact of these effects on patient outcomes. Despite these limitations, using a real‐world patient population that was followed up for a sufficient time post‐NTZ cessation and the inclusion of objective data measurements collected throughout several visits may still allow for the generalizability of results because the sample is representative of patients with MS in our Middle Eastern population. Finally, our study design reflects a more realistic approach to achieving optimal control of MS disease activity, as studies employing fixed washout durations or initiation schedules for alternative therapy may be limited by ethical considerations for patient safety.

## 5 Conclusions

Due to the growing prevalence of MS and its significant social and economic impacts in the Middle East, particularly Lebanon, this research supports reducing the treatment gap following the cessation of NTZ. It also presents evidence of pseudoatrophy and persistent brain inflammation post‐NTZ, suggesting effective DMTs like rituximab could mitigate these effects. Nevertheless, the study advocates for larger multicenter research across the MENA region to evaluate and compare the impact of post‐NTZ DMTs on disease activity and cognitive decline. Additionally, further observational studies on sequential DMTs will enhance the external validity and applicability of these findings across both regional and global MS populations. Finally, further research on identifying predictors of disease reactivation is crucial to enhance MS management in resource‐limited settings, ultimately aiming for improved patient outcomes amid healthcare challenges.

## Author Contributions

**Roba El Zibaoui**: conceptualization, methodology, data curation, formal analysis, writing – original draft, writing – review and editing, visualization, investigation, software, project administration. **Samia J. Khoury**: conceptualization, methodology, data curation, supervision, writing – review and editing, visualization, project administration, formal analysis, investigation, validation. **Nabil K. El Ayoubi**: conceptualization, methodology, data curation, formal analysis, supervision, writing ‐ review and editing, visualization, validation, investigation, project administration.

## Conflicts of Interest

The authors declare no conflicts of interest.

## Peer Review

The peer review history for this article is available at https://publons.com/publon/10.1002/brb3.70904.

## Data Availability

The data that support the findings of this study are available on request from the corresponding author. The data are not publicly available due to privacy or ethical restrictions.
